# Supplementary material for: yama, a mutant allele of Mov10l1, disrupts retrotransposon silencing and piRNA biogenesis
Source: PLoS Genet. 2021 Feb 26;17(2):e1009265. doi: 10.1371/journal.pgen.1009265 (PMC7946307; doi:10.1371/journal.pgen.1009265)
Supplement: S1 Fig — Exon 5 sequence is shown in red (Reference cDNA sequence: NCBI accession number XM_006521556). The mutation is highlighted in green in exon 5 (GTG229GAG ➔ V229E). BseRI site is underlined: GAGGAG(N)10. PCR genotyping primers: Forward primer is highlighted in yellow. Reverse primer is highlighted in magenta. (DOCX) [file pgen.1009265.s001.docx]

The *Mov10l1* *yama* allele sequence

Exon 5

TGCAGGTTTGCATTTCCAGCCTGTGTGGGAGGAACGGGGTGATAGAGGACAGCATCTTCTTCAGCCTGGACTCC

Forward primer GTG BseRI

TTGAAGCTGCCGGAAGGGTACATACCGAGGAGACACGACATTGTCAATGCTGTGGTTGAGGAGAGCAGCCAGTC

Intron 5

ATGCTACATCTGGAGAGCACTGTGCATGACCCCTGTGAAGAGGTGGTATCCAAACAATTCTTTGGAAATAAAAG

GGACAGTTTTAAGCATATTTTAAAGAGTTGTAGTCAAGTGCTGTCTACCTTTTCAGTCAGTCACGGGTTGTGTA

TACGATTCTGGTTCCACTAGATCATACCACCAGGCG

Reverse primer

WT = T

Mutant = A
