# Supplementary figures and images for: yama, a mutant allele of Mov10l1, disrupts retrotransposon silencing and piRNA biogenesis
Source: PLoS Genet. 2021 Feb 26;17(2):e1009265. doi: 10.1371/journal.pgen.1009265 (PMC7946307; doi:10.1371/journal.pgen.1009265)

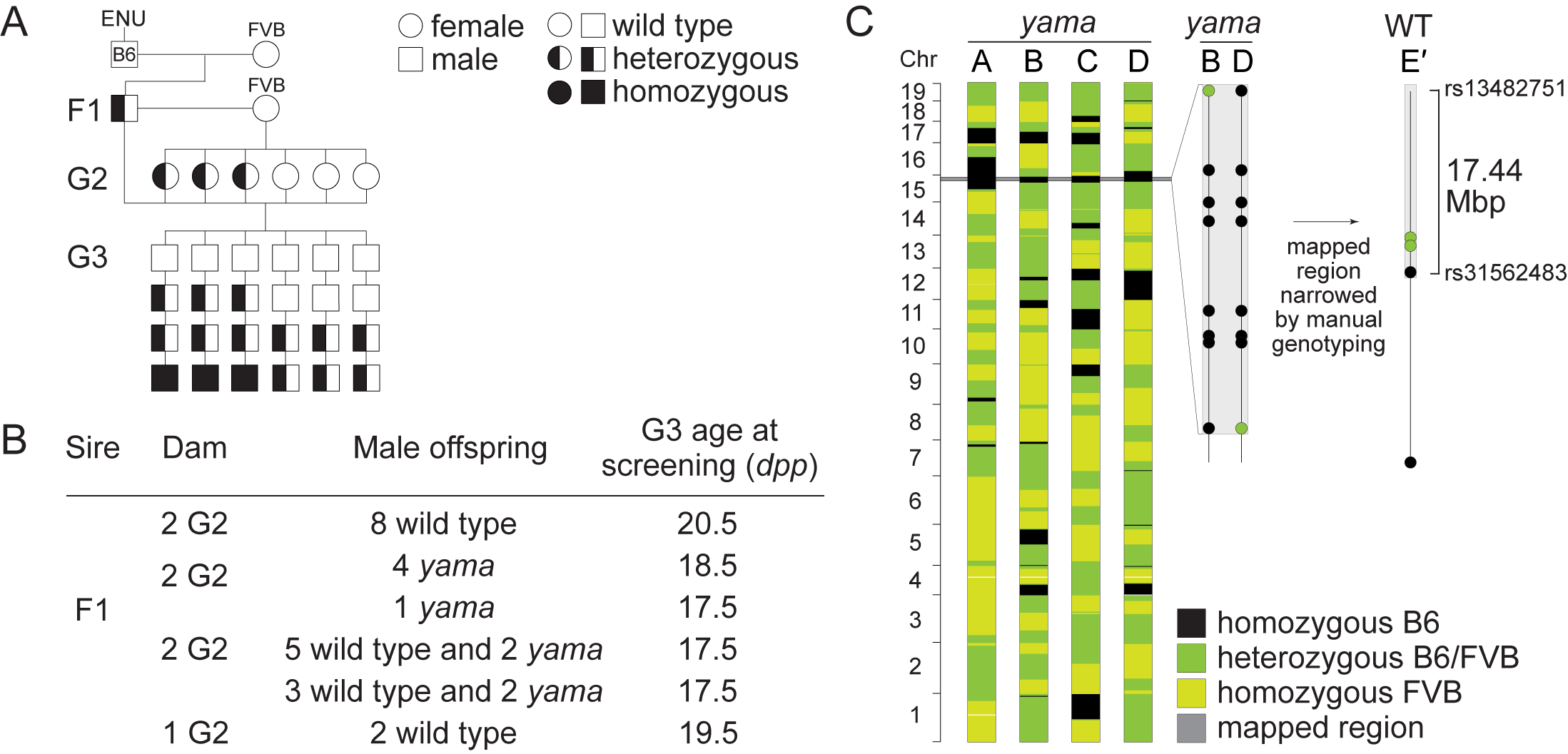

Supplement: S2 Fig — Histology of testes from 4-month and 7-month-old males. Asterisks indicate tubules with Sertoli-cell-only phenotype or severe depletion of germ cells. Scale bar, 50 μm. (TIF) [file pgen.1009265.s002.tif]

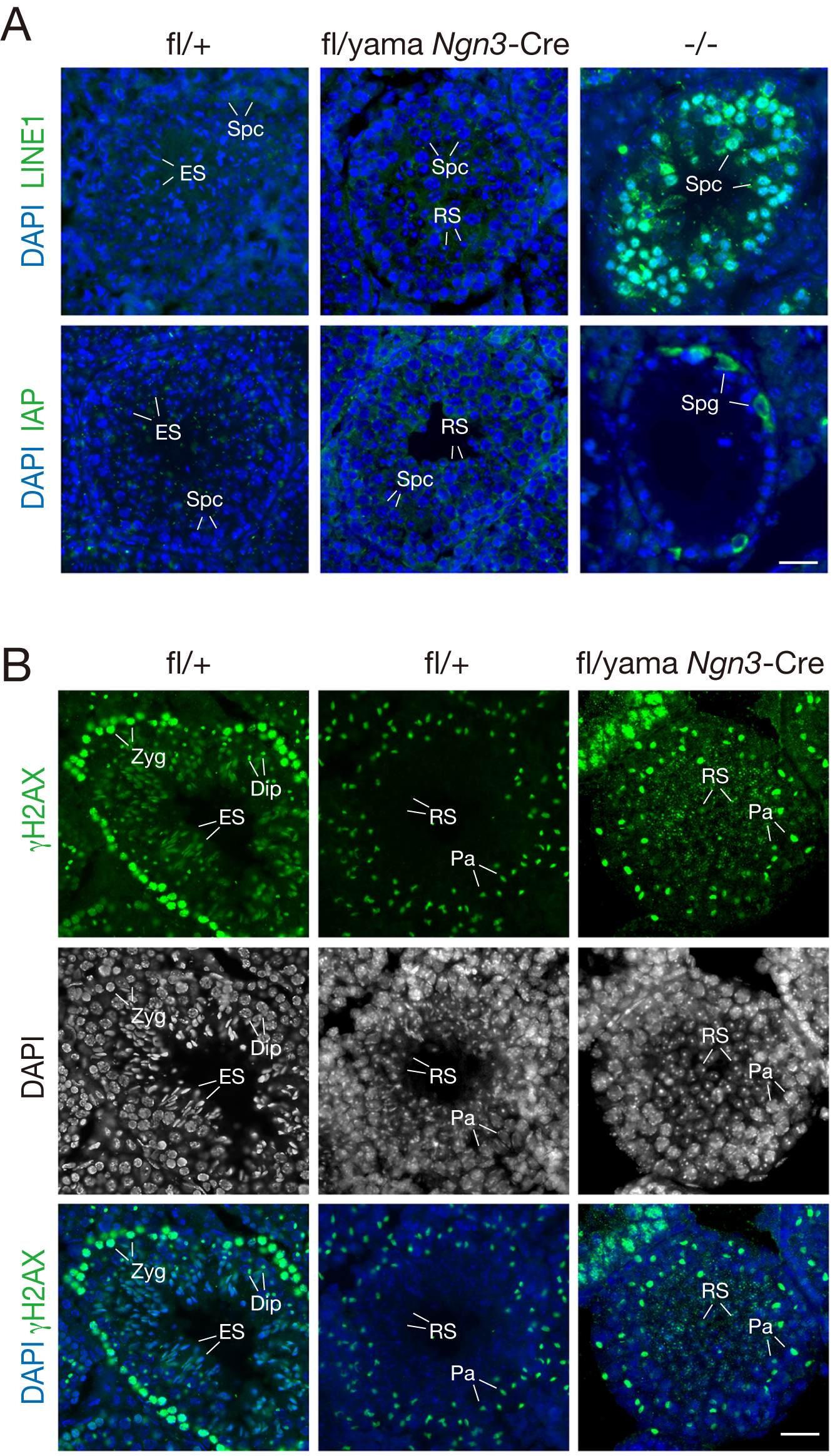

Supplement: S3 Fig — (A) Sections of testes from 6-week-old wild-type and Mov10l1fl/yama Ngn3-Cre males were immunostained with anti-LINE1 and anti-IAP antibodies. Mov10l1-/- (knockout) testis serves as a positive control [41]. (B) Presence of γH2AX in round spermatids from 6-week-old Mov10l1fl/yama Ngn3-Cre testes (right panels). Elongating spermatids in Mov10l1fl/+ (control) stage XI tubules are γH2AX-positive (left panels). Round spermatids in Mov10l1fl/+ (control) early stage (before IX) tubules are γH2AX-negative (middle panels) but round spermatids in Mov10l1fl/yama Ngn3-Cre testes (right panels) are γH2AX-positive. Abbreviations: Spg, spermatogonia; Spc, spermatocytes; RS, round spermatids; ES, elongating spermatids; Zyg, zygotene spermatocytes; Pa, pachytene spermatocytes; Dip, diplotene spermatocytes. Scale bars, 25 μm. (TIF) [file pgen.1009265.s003.tif]

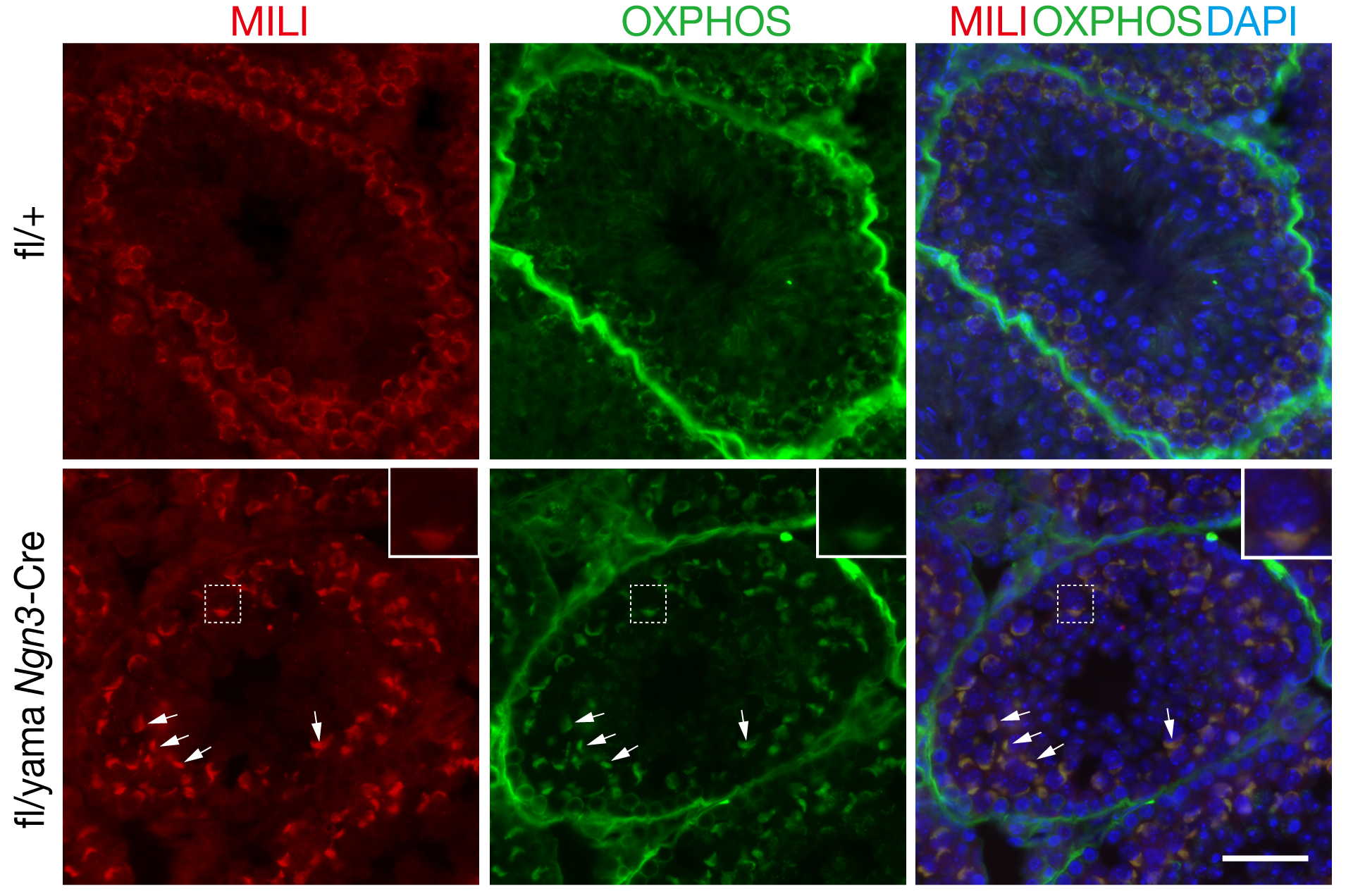

Supplement: S4 Fig — Testis sections from P60 Mov10l1fl/+ (control) and Mov10l1fl/yama Ngn3-Cre mice were immunostained with anti-MILI antibody and OXPHOS. OXPHOS is a cocktail of five monoclonal antibodies against mitochondrial proteins (S2 Table). MILI and mitochondria colocalize but are distributed throughout the cytoplasm in Mov10l1fl/+ pachytene spermatocytes. However, only polar aggregates in Mov10l1fl/yama Ngn3-Cre pachytene spermatocytes are OXOPHOS-positive. Representative polar aggregates are indicated by arrows. Inset shows an enlarged view of the boxed spermatocyte. Scale bar, 25 μm. (TIF) [file pgen.1009265.s004.tif]
