# Supplementary material for: yama, a mutant allele of Mov10l1, disrupts retrotransposon silencing and piRNA biogenesis
Source: PLoS Genet. 2021 Feb 26;17(2):e1009265. doi: 10.1371/journal.pgen.1009265 (PMC7946307; doi:10.1371/journal.pgen.1009265)
Supplement: S1 Table — (DOCX) [file pgen.1009265.s005.docx]

**S1 Table. Primary and secondary antibodies.**

| Antibody | Host | Producer | Cat. No/reference | Dilution | |
| --- | --- | --- | --- | --- | --- |
|  |  |  |  | WB | IF |
| SYCP1 | Rabbit | Abcam | ab15090 |  | 1:100 |
| SYCP3 | Mouse | Abcam | ab97672 |  | 1:200 |
| LINE1 | Rabbit | Gift from Ramesh Pillai | [1] | 1:1000 | 1:200 |
| IAP | Rabbit | Gift from Bryan Cullen | [2] | 1:2000 | 1:200 |
| TDRD1 | Rabbit | Gift from Shinichiro Chuma | [3] | 1:2000 | 1:200 |
| MYC | Mouse | Clontech | 631206 | 1:2000 |  |
| HA | Mouse | Roche | 12CA5 | 1:5000 |  |
| ACTB | Mouse | Sigma | A5441 | 1:5000 |  |
| V5 | Mouse | Invitrogen | R960-25 | 1:2000 |  |
| MILI | Rabbit | Abcam | ab36764 | 1:2000 | 1:200 |
| MIWI | Rabbit | Abcam | ab12337 | 1:2000 | 1:200 |
| MOV10L1 | Rabbit | Custom-made | [4] | 1:500 | 1:100 |
| PLD6 | Mouse | MBL | M207-3 | 1:1000 | 1:100 |
| γH2AX | Mouse | Millipore | 05-636 |  | 1:500 |
| SP10 (ACRV1) | Guinea pig | Prabhakara Reddi | [5] |  | 1:100 |
| OXPHOS | Mouse | Abcam | ab110413 |  | 1:300 |
| Anti-rabbit IgG Fluorescein | Goat | Vector Laboratories | FI-1000 |  | 1:200 |
| Anti-mouse IgG Texas Red | Horse | Vector Laboratories | TI-1000 |  | 1:200 |
| Anti-mouse IgG,  HRP-linked | Horse | Cell Signaling | 7076S | 1:5000 |  |
| Anti-rabbit IgG,  HRP-linked | Goat | Cell Signaling | 7074S | 1:5000 |  |

**References**

1. Wenda JM, Homolka D, Yang Z, Spinelli P, Sachidanandam R, Pandey RR, et al. (2017) Distinct roles of RNA helicases MVH and TDRD9 in PIWI slicing-triggered mammalian piRNA biogenesis and function. Dev Cell 41: 623-637.e9.

2. Bogerd HP, Wiegand HL, Doehle BP, Lueders KK, Cullen BR. (2006) APOBEC3A and APOBEC3B are potent inhibitors of LTR-retrotransposon function in human cells. Nucleic Acids Res 34: 89-95.

3. Chuma S, Hiyoshi M, Yamamoto A, Hosokawa M, Takamune K, Nakatsuji N. (2003) Mouse tudor repeat-1 (MTR-1) is a novel component of chromatoid bodies/nuages in male germ cells and forms a complex with snRNPs. Mech Dev 120: 979-990.

4. Zheng K, Xiol J, Reuter M, Eckardt S, Leu NA, McLaughlin KJ, et al. (2010) Mouse MOV10L1 associates with piwi proteins and is an essential component of the piwi-interacting RNA (piRNA) pathway. Proc Natl Acad Sci U S A 107: 11841-11846.

5. Reddi PP, Naaby-Hansen S, Aguolnik I, Tsai JY, Silver LM, Flickinger CJ, et al. (1995) Complementary deoxyribonucleic acid cloning and characterization of mSP-10: The mouse homologue of human acrosomal protein SP-10. Biol Reprod 53: 873-881.
