# Supplementary material for: yama, a mutant allele of Mov10l1, disrupts retrotransposon silencing and piRNA biogenesis
Source: PLoS Genet. 2021 Feb 26;17(2):e1009265. doi: 10.1371/journal.pgen.1009265 (PMC7946307; doi:10.1371/journal.pgen.1009265)
Supplement: S2 Table — (DOCX) [file pgen.1009265.s006.docx]

**S2 Table. Real-time PCR primers**

| Target | Primer name | Primer sequence | Product (bp) |
| --- | --- | --- | --- |
| pre-piR1 | pre-piR1-F | GTTAGCGAAGGACATTATTCTAACC | 501 |
|  | pre-piR1-R | TGACATGAACACAGGTGCTCAGAT |  |
| pre-piR2 | pre-piR2-F | CTATGCTTATGATGGCATTGGAGAG | 530 |
|  | pre-piR2-R | TTCCAGTTCAACAGGGACACGGGAC |  |
| pre-piLR | pre-piRNA2-F | GTGAAGCTAAGGATGCTGGGATAG | 413 |
|  | pre-piRNA2-R | ACAGGATGTCCCCTGAAATCAGTC |  |
| Line1 | Line1-F | GAGAACATCGGCACAACAATC | 762 |
|  | Line1-R | TTTATTGGCGAGTTGAGACCA |  |
| pri-let7g  IAP | pri-let-7gF | GTACGGTGTGGACCTCATCA | 137  330 |
|  | pri-let-7Gr  IAP-F  IAP-R | TCTTGCTGTGTCCAGGAAAG  GCACCCTCAAAGCCTATCTTAT  TCCCTTGGTCAGTCTGGATTT |  |
| *Actb* | Actin-F | AGAAGAGCTATGAGCTGCCT | 382 |
|  | Actin-R | TCATCGTACTCCTGCTTGCT |  |
