# Supplementary material for: yama, a mutant allele of Mov10l1, disrupts retrotransposon silencing and piRNA biogenesis
Source: PLoS Genet. 2021 Feb 26;17(2):e1009265. doi: 10.1371/journal.pgen.1009265 (PMC7946307; doi:10.1371/journal.pgen.1009265)
Supplement: Attachment — Submitted filename: ResponseToReviews.pdf [file pgen.1009265.s007.pdf]

Paula E. Cohen
Associate Editor
PLOS Genetics

Gregory Barsh
Editor-in-Chief
PLOS Genetics

Re: PGENETICS-D-20-01770

Dear Drs. Cohen and Barsh,

We'd like to thank all the reviewers for their comments. We have addressed all the concerns with new data and text revisions. We have made additional minor text revisions to improve clarity.

We have added two new supplementary figures:
S2 Fig. Progressive depletion of germ cells in aged *Mov10l1*$^{yama/yama}$ males.
S4 Fig. Colocalization of mitochondria with polar aggregates in pachytene spermatocytes from *Mov10l1*$^{fl/yama}$ *Ngn3*-Cre testes.

Please note that Devanshi Jain and Scott Keeney are switched in the order of authors. All authors have agreed with this change.

A marked up copy of the manuscript has also been uploaded.

Below is our point-by-point response to the reviewers' comments.

Reviewer #1

Thank you for submitting this manuscript. The experiments described within aim to characterize the poorly understood function of the N-Terminal domain of MOV10L1, a germ cell specific RNA helicase essential for piRNA biogenesis. The authors describe a single amino acid substitution in the N-Terminal region they call "yama", that causes meiotic arrest and male sterility. To dissect Mov10L's role in pre-pachytene and pachytene piRNAs, the authors cleverly designed a conditional mouse model shifting the mutation to the later piRNA biogenesis stage, also resulting in meiotic arrest but with distinct difference. This work provides more mechanistic understanding of both pre-pachytene and pachytene biogenesis and provides a functional link between MOV10L and PLD6.

MAJOR:

Fig2D: How is the SYCP1/3 defect linked to the MOV10L deficiency? Is this simply a readout? If the defect is interpreted as a block or delay in meiosis, then a timecourse showing the previous stages would be expected to be identical at zygotene, just a stall. Is this correct? Otherwise, the term "zygotene-like" is appropriate but then also needs to be supported. If HET and KO zygotene are identical, then the interpretation needs to be adjusted slightly. If this is a blockade, would you expect an increase in Zygotene presenting cells? Or is the implication that these zygotene-like cells are directly shuttled into an apoptotic pathway? A quantification of zygotene accumulation, linked to the increased TUNEL staining in 2F would be a decent link/bridge for this interpretation. Perhaps a simple ATM or ATR staining on spreads would support the checkpoint suggestion in line 140.
Response: The synapsis defect (SYCP1/3) is a readout, which is a common characteristic of piRNA pathway mutants such as *Mili*, *Miwi2*, *Mov10l1*, *Mael*, *Tdrd1*, *Pld6*, etc. Thus, it is not unique to the

yama mutant. The *yama* mutant males exhibit meiotic arrest (blockade). We examined the testis histology at different developmental timepoints: P21 (first wave of spermatogenesis or juvenile wave), P28 (first wave), and P60 (adult) (Fig. 2D). The *yama*/*yama* mutant testes exhibit similar meiotic arrest at P21, P28, and P60, whereas the heterozygous control testes develop more advanced germ cells (elongated spermatids) with age as expected. The increased apoptosis supports that the zygotene-like spermatocytes are eventually eliminated via apoptosis and thus do not accumulate. Since the increased apoptosis of spermatocytes is common in nearly all piRNA mutants and thus is not unique to the *yama* mutant, we believe that further analysis of the meiotic checkpoint activation is interesting but beyond the scope of the current study.

Fig2G and H: The stability hypothesis was addressed nicely, but can you elaborate more on why such a noticeable difference in abundance is seen between p21 and p60? Is the "first wave" intrinsically distinct in this particular analysis? Is there a different proportion of spermatocytes between these stages? I don't think so but please correct me. Presumably pachytene is depleted in both ages similarly. Could another explanation be that the defects become more pronounced over time? Would a 90 or 120 day mouse have even less? I would consider exploring this briefly to link this phenotype to age related loss of fertility in humans. Could you also confirm the antibody was not raised against this particular region (silly control but worth asking)? Otherwise I would imagine there are in vivo post translational modification and regulation explaining this.
<u>Response</u>: We agree on age as being a possible explanation of lower abundance at P60. It is known that mutants with meiotic arrest display more severe phenotypes (progressive loss of germ cells) with age. This is indeed the case for yama mutant. We have included a new supplementary figure (S2 Fig) to show 4-month and 7-month-old *Mov10l1*$^{yama/yama}$ testis histology. While Sertoli-cell-only tubules were not observed in P60 or younger testes, tubules with Sertoli-cell-only or a severe loss of germ cells appeared in 4-month-old (25% tubules) and 7-month-old (71% tubules) *Mov10l1*$^{yama/yama}$ testes, showing a progressive loss of germ cells with age (S2 Fig). We have added this result (Lines 136-139). The antibody was raised against the N-terminal region (aa 1-101) of MOV10L1, which does not contain the V229 residue. We have added this information in Fig. 2 legend.

Fig3A and B: Does the localization of upregulated LINE1 (spermatocytes) and IAP (Spermatogonia) correspond properly with what is expected from Mov10L (yama) defect? The Zheng 2010 references suggests similar pattern, but can you comment on the severity? Is this a phenocopy? If so, how much of the null mutation can you attribute to the N-terminal mutation and previous studies describing the helicase domain?
<u>Response:</u> This upregulation of LINE1 and IAP at different developmental stages is expected in the *yama* mutant, since the primary defect in this mutant is the failure of piRNA biogenesis, which also occurs in the MOV10L1 helicase domain deletion mutant. Our data suggest that this phenotype is a phenocopy. Both mutants lack piRNAs. The severity appears to be similar based on P10 qRT-PCR data. This cell type-specific de-repression of LINE1 and IAP in mutant testis is also observed in other (*Mili* and *Tex15*) mutants with retrotransposon de-repression, because LINE1 and IAP repressions require additional different mechanisms in different types of germ cells. We have expanded this explanation in the Results (Lines 186-189).

Fig4: The previous set of experiments used HET vs KO. The following experiments use WT vs fl/yama,Cre+. Is the reason for this the fl/yama,Cre- animal is compromised in some way? I might just be confused. I think a Supp figure diagram would really help me understand.
<u>Response:</u> The *Mov10l1*$^{yama/+}$ (heterozygous) and *Mov10l1*$^{+/-}$ mice were fertile with no observable spermatogenic defects. The WT control in Fig 4 is *Mov10l1*$^{fl/+}$, Cre negative. These control mice were littermates of mutants. We apologize for oversimplification in labeling. We have changed WT to fl/+ in Fig 4.

Figure 6: I wonder if the persistent binding is simply technical? Have you tried using the MYC or HA antibodies for exposing the protein? Maybe an un-tagged prey? In Vitro CO-IP are something voodoo. It is also the wrong species and the wrong cell type. Are you in possession of a reliable mouse cell line that can be transfected? I can only think of ES Cells or pMEFs at the moment. The in vivo testis data is more important to me in any case.
Response: All the Western blotting analyses were performed with either anti-MYC, anti-HA, or anti-V5 antibody. We have added this clarification in Figure 6 legend. We have tried NIH3T3 cells, however, the transfection efficiency for some proteins was very low in 3T3 cells. HEK293 cells give consistently high transfection, which is important for these types of interaction and domain-mapping studies.

Model: I liked the connection between the phenotype and the aggregates seen in figure 5, but I feel like there wasn't enough exploration into this observation to generate the model. It is left mostly speculative at this point. The model shows mitochondrial involvement but no experiments were done to make that link. The study and model suggests an accumulation of piRNA precursor transcripts and the aggregates suggest this is happening at or around the mitochondria. The mitochondria normally exist all throughout cells (unless germ cells are different) so you could imagine a mitochondrial aggregate as well. I think to strengthen this link either RNA-FISH (piRNA precursor), or a co-localization study of the aggregates and a mitochondrial marker would convincing. It would also provide a possible link to metabolic disfunction, since a clumped up mitochondria is an unhappy mitochondria. Work by David Chan at Caltech might be worth a look.
Response: We have examined the localization of mitochondria with OXPHOS. OXPHOS is a cocktail of five monoclonal antibodies against mitochondrial proteins. Mitochondria are no longer distributed throughout the cytoplasm, but instead are clustered with polar aggregates in mutant spermatocytes. We have included this new data in a new supplementary figure (Fig S4). The results are described in lines 261-265. Several piRNA pathway proteins such as PLD6, PNLDC1, and TDRKH are mitochondrial outer membrane proteins. Mitochondria-containing protein extract is required for in vitro piRNA biogenesis. Whether mitochondria play a structural or metabolic role in piRNA biogenesis is not clear. We have expanded discussion on these points (Lines 358-360).

MINOR:
-In general, can you provide explanation for experiments where one of the p10, p21 and p60 timepoints are not presented? There seems to be a bit of jumping around in their inclusion throughout the manuscript.
Response: Most studies were done with P21 (juvenile) and P60 (adult). The main reason is that we would like to analyze both the first wave and adult waves of spermatogenesis. In fact, the mutant phenotype did not differ between P21 and P60. Juvenile testes (P21) were used for co-IP analysis, because the control (het) testis was closer (still more than) in germ cell content to the mutant, unlike the dramatic size difference between P60 het and P60 mutant testes. P10 testes was also used for LINE1 and IAP analysis, because they contained spermatogonia and pre-leptotene spermatocytes but no other germ cells. We have added these explanations/clarifications in the revised manuscript where appropriate.

-Line 51: How conserved are mouse and human across the entire length of MOV10L (overall % conservation)?
Response: The protein sequence identity between mouse and human MOV10L1 is 77%.

-Line 60: Typo, should it be PIWIL4?
Response: corrected.

-Line 96: Very cool!
-Line 106: Present pdf makes it very hard to see details in A.
<u>Response:</u> We have now increased the font sizes in Fig. 1. The resolution in the PDF was reduced in comparison with the original tiff file, it was partly due to size reduction upon pdf conversion at the journal.

-Line 107: Can you elaborate at least one sentence on "meiotic defects"? What is solely based on SYCP3/yH2AX?
<u>Response:</u> The double staining allows for rapid assessment of defects in both synapsis and meiotic recombination. We have added this sentence.

-Line 116: Danio Rerio has an L in position 229. Would a leucine in this position be predicted to be functionally equivalent? Can this be shaded differently, be given a conservation score or simply remove zebrafish? Also, dos this region share ANY similarity to a known domain or is all this space seemingly room to interact with the various PIWI pathway components?
<u>Response</u>: L and V are structurally similar. Both are neutral. Leucine has an extra carbon (CH2). Leucine in this position is expected to be functionally equivalent. The leucine has been shaded differently. The MOV10L1 N-terminal region does not share similarity with known domains or motifs. We have added this sentence in Introduction (Line 91-92).

-Line123: Can you label the type of histological staining on Fig2D? Legends just say "Histology". Presumably H&E.
<u>Response</u>: H&E is now added in figure legend.

-Line160: Please avoid the use of "significant" unless associated with statistics. While obvious from the representative images, there may be regions of the section with similar mis-regulation due to age or other unrelated defects.
<u>Response</u>: changed.

-Line 164: What is used as a baseline in Fig 1D (The reviewer meant 3D)? The Western blot appears blank in WT. Is there a reason why p60 is excluded? Can you add statistics to this observation (het is missing error bars). Also, the IAP background in the WT control lane appears higher than expected. Is this consistent with the literature? Is this possibly a consequence of the mixed background used (or has this been sufficiently backcrossed to make that not a concern?)
<u>Response</u>: LINE1 appears to be blank in WT relative to the mutant, because LINE1 ORF1 protein level is very high in the mutant when equal amounts of total proteins are loaded. P60 was assessed by both IF (Fig 3A-B) and WB (Fig 3C). Therefore, we felt it unnecessary to do qRT-PCR at P60. We have added statistics to Fig 3D and its legend. There are IAP signals in the WT. All the publications appear to have used the same IAP antibody, which does give higher background in WT. Importantly, the IAP GAG protein abundance increased dramatically in the mutant, which is consistent with IF and qRT-PCR results. We don't think that it is due to the mixed genetic background.

-Line 165: The statement isn't completely supported. You cannot say they are not silenced without a comparison to a system where retrotransposon regulation is completely shut down. You can revise this to say something like "de-repressed".
<u>Response</u>: Thank you! Corrected.

-Line 172: For 3F, The staining of MIWI in p60 is a bit more unusual than described. Is this representative? The surrounding tissues seems fairly positive for signal (I see a bit of this in 3E as

well). Also, there is low level staining and two spots that might be nuclear (are they)? If this is not representative, please consider a different image. Do you have a lower magnification?
Response: the P60 image was overexposed, as MIWI signal was missing in spermatocytes. The surrounding interstitial signal was due to overexposed autofluorescence from Leydig cells. The P60 mutant image in Fig 3F has been replaced with a more representative image.

-Line 172: Can you add a sentence as to the significance of this finding? If it is totally expected, can you say how much of your expectations were matched? Should this totally phenocopy the null mutant?
Response: We have revised this sentence for clarity. The absence of MIWI in yama/yama testis is totally expected, because MIWI is normally expressed in pachytene spermatocytes, which are missing in the mutant testis. This result is not important but included for comparison with MILI and completeness.

-Line 194: I think the labeling throughout figure 4 is a little inconsistent. Sometimes NGN3 is shown. Something is it just “Cre”. Also, the control in 4D is a fl/+ mouse. Is this a typo or are there different controls throughout this figure?
Response: corrected.

-Line 199: Please provide error bars and statistics (student’s t-test. Show SEM assuming this was done in biological replicates). The Pre-piLR significance is unclear.
Response: It is related to Fig. 4E. The controls were set at 1 and thus do not have error bars. We have added statistics. The increase of Pre-piLR in mutant is statistically significant.

-Line205: Is the lack of LINE1 expression accurately controlled? I would consider evaluating whether the specific LINE1 family primers actually corresponds to an intact and functional ORF1p generating LINE1 subfamily. You have the perfect control actually. Can you show an increase in LINE and IAP through qPCR on the testes showing in Figure 3A and 3B (this would be nice to include in figure 3 anyway).
Response: This comment is related to Fig. S3A (formerly Fig S2A). We did qRT-PCR of LINE1 in fl/yama Ngn3-Cre testis in Fig. 4E and there was no increase in the mutant. We did not do qRT-PCR of IAP. We did have a positive control for IF of both LINE1 and IAP using *Mov10l1*$^{-/-}$ testis (Fig S3A). The LINE1 primers map to ORF1 and ORF2 conserved sequences. We have not been able to map them to a particular LINE1 subfamily.

Line 217: S2b legends are written in a slightly confusing way. Please consider revising and confirm they properly correspond to image.
Response: We have revised the legend (now Fig S3B) for clarity.

-Line 227: Is there any appreciable difference in MOV10L1 localization of abundance in any of the mutants from this analysis?
Response: we did not observe changes in MOV10L1^{V229E} localization.

-Line 244: I think it should be (Fig. 5J) to show abolished association.
Response: Corrected. Thank you for catching this error!

-Line 261: Very minor, but please consider moving the image of 6h earlier in the image so that the figure citations are in order. (Turn 6H into 6F)
Response: We prefer to keep it as is. We agree that the citation of 6H is ahead of 6F and 6G. We think that 6H serves as a summary and it is more appropriate to be the last panel.

-Line 628: hard to understand, please consider revising. Could “phenotypically WT” technically be mutated at this region?
Response: It has been changed to “phenotypically normal”.

Reviewer #2
This manuscript by Guan et al., identifies a single amino acid point mutation in the piRNA biogenesis factor called MOV10L1. Previous studies have shown that MOV10L1 is RNA helicase, and this RNA helicase activity residing in the C-terminal part of the protein is absolutely essential for piRNA biogenesis, and as a consequence is required for transposon silencing in the mouse male germline, and is required for male fertility. In this study, they identify a point mutation in the highly conserved N-terminal region, whose functions are not known. The authors demonstrate that the mutant protein is expressed, albeit at reduced levels, but is unable to support the normal functions of the protein. Using some clever genetics approaches, they examine the impact of the mutation in two different developmental windows during spermatogenesis, when two distinct populations of piRNAs are expressed.
This is an elegant study that combines genetics and biochemistry, and for the first time sheds light on the non-catalytic role of MOV10L1 in piRNA biogenesis. It uses its N-terminal domain to assemble the piRNA biogenesis machinery, by directly recruiting the endonuclease responsible for 5’ end generation of piRNAs. This finding will have huge implications for our current model of how the complex is assembled. I support its publication.

Minor comments:
Figure 5D: Why is the yama version of Mov10L1 not accumulating in the unique crescent-shaped structures? One would have expected Mov10L1 to be in these structures like MILI, MIWI and TDRD1. Is it because of an antibody issue? A mention of this in the discussion would be good.
Response: The yama version of MOV10L1^{V229E}, like the wild type protein, localizes throughout the cytoplasm but does not accumulate in the polar crescent-shaped structures. We don’t think that this is an antibody issue, as the wild type control showed the expected localization pattern. We have added a new supplementary figure (Fig. S4) to show that the crescent-shaped structures overlap with mitochondria. PLD6 is a mitochondria protein. The reduced interaction between MOV10L1^{V229E} and PLD6 is likely to cause or contribute to a failure in recruitment of MOV10L1 to mitochondria by PLD6. We have added this to discussion (Line 363-365).

Figure 5D: It is interesting that yama mutant Mov10L1 fails to interact with PLD6. It would be interesting to localize PLD6 and see if it is also in the crescent structures. I would imagine that it should not be there, as it fails to interact with Mov10L1. I am not sure if suitable antibodies are available, but if this data can be obtained, it could be added.
Response: We have tried IF but did not see signals in pachytene spermatocytes with the PLD6 antibody. We have not found reports of endogenous PLD6 immunolocalization in tissues from the literature.

Reviewer #3
In this manuscript the authors report on a new mutation in the Mov10l1 gene, affecting structure of the N-terminal region, for which the function was previously unknown. They find that the mutation causes male infertility, derepression of LINE1 and IAP retrotransposons, and aberrant processing of both pre-pachytene and pachytene piRNAs. An apparent primary cause of the aberrant piRNAs is failure of the mutated protein to bind to PLD6, a key endoribonuclease in the piRNA processing pathway, thereby assigning functional significance to the N-terminal domain of the protein. As a summary evaluation, the manuscript is clearly written, the figures are excellent (gorgeous histology and IF!), the conclusions are supported by the results, and the overall findings are of significant

interest.

This study was based on the phenotype of a recent ENU-induced mutation, “yama,” in the Mov10l1 gene. The gold standard for ENU mutagenesis programs is to prove that the detected mutation is causative of the phenotype (rather than an undetected or under-appreciated regulatory SNP in nearby sequence). The authors provide robust evidence that the yama mutation is Mov10l1 is the sole cause of the phenotype. First, the parameters of homozygous yama infertility phenocopy the original Mov10L1 knockout mutation; second and most definitively, the yama infertility phenotype does not complement a conditional Mov10l1 allele (hets are infertile, revealing MOV10L1 function post-pachynema); and third, biochemical analyses reveal the mutant yama MOV10L1 protein lacks protein interactions (specifically with PLD6) typical of wildtype MOV10L1.

Overall, the manuscript is excellent: the quality of the figures is high and the data strongly support the conclusion of aberrant function of the yama mutant MOV10L1. There are, however, some points that the authors should consider in order to improve clarity:

Fig. 1A Legend (p. 20, line 616): It is stated that 1/8 of the G3 males are expected to be hom, but this should be clarified to state that 1/8 of all individuals are both male and hom (or that ¼ of males are hom).
<u>Response</u>: our originial statement is correct, because all the G2 daughters (half are wild type) were backcrossed to the F1 sire male. We have added this clarification on G2 daughters (Line 704).

Fig. 3B: What are the intensely staining cells in the lower left? These appear to be interstitial cells (???).
<u>Response</u>: The interstitial cells appeared to have a high level of autofluorescence or non-specific IF signal. The image has been replaced.

Fig. 4: It would be nice, though not necessary, to have images of the conditional hom Mov10l1 flox-Ngn3-Cre.
<u>Response</u>: We have added histological images of *Mov10l1*$^{fl/-}$ *Ngn3*-Cre to Fig 4C for comparison.

p. 7, line 187: The word “lied” should be changed to “is the detected missense mutation V229E.”
<u>Response</u>: Changed as suggested. Thank you!

p. 8, lines 214-216: This sentence needs to be clarified; it doesn’t make sense as written, especially since gH2AX typically localizes to spermatocytes.
<u>Response</u>: We have added clarifications. γH2AX is present in elongating spermatids due to topoisomerase activity in addition to the typical presence in spermatocytes.

p. 8, line 226: The localization to one pole does not in itself indicate that the piRNA path is disturbed but does reveal aberrant localization of the components.
<u>Response</u>: revised accordingly (Line 258-259).

Fig. 7B and associated text about the proposed mode of action of MOV10L1 and PLD6: Fig. 7B is helpful in portraying the proposed pathway and yama defect with the exception of lack of clarity with respect to the line labeled “mitochondrion.” This line (and label) is not described in the legend, and although localization of granules and nuage to mitochondrial cement is mentioned in the text, the role of mitochondria and/or the mitochondrial membrane (is that what the gray line is?) per se is not discussed. Indeed, the Discussion would be more impactful with a bit more attention to the still-evolving concepts of nuage, the aberrant polar accumulation of piRNA pathway proteins (but not

MOV10L1) in conditional mutants, and the significance of localization to subcellular domains of mitochondrial cement.
Response: We have expanded Fig. 7B legend. We have also expanded the discussion as suggested (Lines 345-351; 358-360). Mitochondrion has been changed to mitochondrial outer membrane. PLD6 is a mitochondrial outer membrane-associated enzyme. Proteins involved in piRNA biogenesis localize to nuage (also called inter-mitochondrial cement or germ cell granule). Nuage are membraneless electron-dense cytoplasmic condensates. It has been suggested that nuage in germ cells may form via liquid-liquid phase separation. Notably, DDX4, an essential factor for piRNA biogenesis, undergoes phase separation both in vitro and in cells. It is possible that disruption of piRNA production causes abnormal phase separation of evenly distributed multiple nuage, leading to fusion into one large polar aggregate per spermatocyte.

I hope that the revised manuscript is suitable for publication.

Sincerely yours,

Jeremy Wang
